# The Effect of Continuous Selection in KiwiCross^®^ Composite Breed on Breed Ancestry and Productivity Performance

**DOI:** 10.3390/ani15020175

**Published:** 2025-01-10

**Authors:** Mohd Jaafar, Bevin Harris, Heather J. Huson

**Affiliations:** 1 Animal Science Department, Cornell University, Ithaca, NY 14850, USA; azwanj@mardi.gov.my; 2 Livestock Improvement, Private Bag 3016, Hamilton 3240, New Zealand; bevin.harris@lic.co.nz

**Keywords:** crossbred, admixture, production efficiency, artificial selection, breed ancestry, KiwiCross^®^, genetic signature, XP-EHH, ROH, HapFLK

## Abstract

Composite crosses result from the mating of two or more distinct cattle breeds. Breeding performance may improve rapidly using a well-organized composite breeding system and a clear selection index. The KiwiCross^®^ is a popular composite cross in New Zealand, combining Holstein-Friesian (high milk production) and Jersey (high milk fat). Production efficiency (PR), a key selection index, is calculated by dividing milk solids produced by mature live weight. Over decades of genetic improvement, KiwiCross^®^ increased milk production significantly. We hypothesized that certain genomic regions from Holstein-Friesian or Jersey breeds were preserved due to artificial selection based on PR. Analysis of genomic regions using XP-EHH, hapFLK, and ROH haplotype statistics revealed selection signatures on BTA 7 and 20 in both high- and low-performance animals, with distinct regions linked to Holstein-Friesian and Jersey ancestry. Our findings suggest that selection acted on different genomic regions across generations and that preserving key ancestry-specific haplotypes is crucial for maintaining performance in composite breeds. Breeders must recognize that selection for specific traits can alter allele frequencies and lead to the loss of beneficial breed-specific haplotypes over time.

## Simple Summary

The study investigates how continuous selective breeding influenced the genetic composition and performance of the KiwiCross^®^ composite breed, a hybrid of Holstein-Friesian and Jersey cattle known for its high milk production and quality. By examining thousands of animals, we aimed to identify specific regions in the cattle genome that contribute to enhanced productivity, such as increased milk yield and efficiency. The findings suggest that targeted breeding practices can preserve beneficial genetic traits from both parent breeds, improving performance over successive generations. Specific regions of the genome under trait selection can be traced back to specific parental breeds and highlighted for genomic improvement and selection programs. However, the study also highlights the risk of losing genetic diversity when more emphasis is placed on one parent breed over others, which could reduce the overall health and sustainability of the breed. These insights are valuable for dairy farmers and breeders seeking to optimize cattle production while maintaining long-term genetic health, offering a pathway to more sustainable and efficient agricultural practices.

## 1. Introduction

### 1.1. Crossbreeding

Composite crossbreeding is an alternative to traditional line or straight breeding. Depending on the number of animals and breeds available, a crossbred animal is created by crossing multiple different breeds or strains of cattle [1]. The resulting breed is a combination, or “composite”, of the different breeds or strains used in crossbreeding. The goal is to create a breed that combines the best traits from each parent breed that is well-suited to the area’s specific environmental and management conditions [2,3]. Surprisingly, the most common industry examples of dairy composites are now known as traditional dairy purebred. This includes Guernsey and Jersey, which developed from crosses between Brittany and Normandy cattle [4,5,6] and the largest dairy breed, Holstein-Friesian, developed from black animals from Batavia (present-day Germany) and white animals from Friesland (present-day Holland) [6,7]. However, after undergoing ongoing selection for desired features such as milk production for decades, these composites are now treated as dairy purebred breeds globally [6]. Similarly, several efforts have been made to combine modern dairy breeds with tropical cattle to incorporate traits of heat tolerance and disease resistance for improved performance in tropical conditions. These tropically adapted cattle, typically Zebu cattle (*B. indicus*), include Gir/Gyr and Red Sindhi, with their thicker skin, resulting in heat tolerance and resistance to insect-borne diseases [8,9] which are crossed with dairy breeds.

In general, establishing composite breeds has two significant advantages, including capitalizing on the relative strengths of existing breeds [10] and generating hybrid vigor. Due to each breed having its own advantages and disadvantages, any single breed is unlikely to be appropriate for all production scenarios. Thus, through composite breeds, the weaknesses of one breed can be offset with the virtues of another, resulting in a new composite breed tailored to specific natural and managerial situations [10,11]. Heterosis or hybrid vigor is an additional performance benefit obtained with a crossbred animal over and above the mean performance of the two parent breeds. In traditional crossbreeding, the heterosis effect may decrease depending on offspring generation and crossbreeding strategy. Thus, multiple breeds may be used to maintain hybrid vigor, but the more breeds used tends to complicate management and incur extra cost to the producer [12].

### 1.2. KiwiCross^®^ Background

The offspring of composite crossbreeding will show a combination of genetic improvements from the parental breeds that increase heterozygosity and mitigate inbreeding depression, both additive and non-additive [13]. Dairy producers can use unique crossbreeding systems to establish a sustainable and profitable system for their farms to maximize breeding goals, environmental fitness, and resources. According to several studies, well-planned breeding programs for Holstein-crossed composite breeds significantly boosted fat plus protein output, conception rates, survival rates, and the number of days cows were open compared to purebred Holstein cows [14,15]. This is supported by the widespread use of the composite breed known as the KiwiCross^®^, which comprises over 50% of the cow population nationwide in New Zealand [16]. The breed was developed from the crossing of two breeds: Holstein-Friesian, superior for its milk yield, and Jersey, which excels in its milk fat content [17]. Hardiness and disease resistance, crucial in New Zealand’s grazing environments, were factors selected for in producing KiwiCross^®^ cattle [18]. The resulting composite animals are mated back with either purebred Holstein-Friesian or Jersey or other KiwiCross^®^ as desired. As a result of their continuous genetic improvement starting from the 1950s, modern KiwiCross^®^ produce 378 kg more milk and 28.7 kg more milk solids than the foundational parental average [16]. One of the selection indexes that the Livestock Improvement Company (LIC) uses is that of production efficiency (PR), which can be defined as milk solids production (kg) divided by mature live weight (kg) [16]. Historically, the Holstein-Friesian breed has been known to be superior in both milk production and mature weight [10,19,20,21]. However, the goal for the PR index is to produce a composite breed that has increased production with minimal increase in mature liveweight. By incorporating the Jersey breed, known to have a smaller stature compared to Holstein-Friesian, body-size is balanced with production [22].

### 1.3. Knowledge-Gap and Hypothesis

While the breeding strategy of crossbreeding is well-established, the impact of crossbreeding, particularly on the genome architecture, is not well characterized. Crossbreeding is known to break up linkage disequilibrium (LD) between SNPs and causative mutations. Thus, examining haplotype selection signatures and genomic breed composition in composite breeds can allow us to better comprehend the genetic consequences of traits under selection and the inheritance of loci in crossbreeding systems [23]. We anticipated that the continuous artificial selection using the PR trait would have a significant impact on genome architecture, as artificial selection pressure will play an important role in maintaining certain genomic haplotypes influencing desired ancestry-specific characteristics. We aimed to map these genomic regions or haplotypes providing a selection advantage back to their historical ancestry to identify the impact of ancestry on performance and genome architecture [24]. This allows us to identify ancestry-specific haplotypes in the composite KiwiCross^®^ that are important for performance and may be used to improve genetic prediction accuracy in the future.

Therefore, the goal of this study was to identify the trend of haplotype selection signatures between high and low PR performance levels in the composite breed KiwiCross^®^. We also sought to compare the selection signature across generations of the composite breed KiwiCross^®^. We expected to observe different regions selected across birth year and performance levels and sought to map these regions or haplotypes back to the parental breeds to identify their breed origin. Identifying these ancestry-specific regions or haplotypes in composite crossbred cattle could aid dairy producers and researchers in efficiently optimizing breeding plans by increasing the accuracy of genomic selection to maximize the benefits of heterosis. Furthermore, various studies suggest that using haplotypes rather than single nucleotide polymorphism (SNP) genotypes usually leads to higher prediction accuracies. Our study aims to identify haplotypes associated with PR performance [25,26]. This may result from haplotype blocks having more SNPs in higher LD, including causative mutations, compared to an individual SNP, which may only explain a portion of the variation.

## 2. Materials and Methods

**Figure 1:**
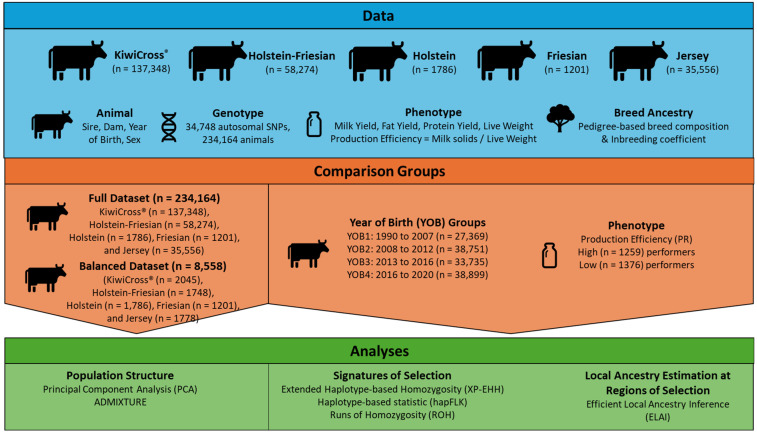
Schematic of the project materials and methodology.

Figure 1 shows a schematic of the project data, datasets assessed, and the analyses used.

### 2.1. Animals

The animal genotypes used in this study were sourced from LIC, Hamilton, New Zealand, which include KiwiCross^®^ (*n* = 137,348), local Holstein-Friesian (*n* = 58,274), Holstein (*n* = 1786), Friesian (*n* = 1201), and Jersey (*n* = 35,556) animals. Breed and pedigree data were extracted from the DairyNZ national database. Using all the available genotype information, parent verification and discovery were undertaken to ensure the pedigree’s accuracy. The breed proportions of the founder animals were dropped down the pedigree. Any animal where the dropped-down breed proportions had a discrepancy greater than 0.25 with the recorded breed proportions was removed. Overall, less than 5% of the initial data were removed. In all cases, the animals removed had one or both parents missing or the parents were not genotyped. This pedigree information was then used to ascertain the proportion of each animal’s breed composition to define animals within each breed population. Purebred animals were regarded as having more than 7/8 breed composition of that breed, whereas composite breed animals include a combination of Holstein, Friesian, and Jersey proportions that are each less than 7/8. As the introduction describes, the composite breed, KiwiCross^®^, is produced through an open mating system where animals are mated to either purebred lineages or another KiwiCross^®^ as desired, resulting in different levels of breed composition in the population. This dataset includes Holstein-Friesian and Jersey breeds that were used as the parental source in developing the composite KiwiCross^®^ and purebred Holstein and Friesian that were not directly related to the KiwiCross^®^. The purebred Holstein and Friesian were included to observe whether the ancestral origins of genomic regions could be mapped back to Holstein or Friesian within the KiwiCross^®^ since the Holstein-Friesian population is similarly a composite breed by origin. The Holstein and Friesian in this dataset are from New Zealand and represent animals from the mid 1900s, prior to KiwiCross^®^ development (LIC, Hamilton, New Zealand). The BovineSNP50 DNA Analysis BeadChip was used to collect genotype information for all animals examined (Illumina, San Diego, CA, USA). Genotype data are supported by measures of four performance traits (milk yield, fat yield, protein yield, and live weight). Other metadata include sire and dam information, birth year (1990–2021), sex, and a pedigree inbreeding coefficient.

### 2.2. Quality Control of Genotypes

The quality of each genotype was evaluated using SNP and Variation Suite (SVS) v8.9.1 (Golden Helix, Inc., Bozeman, MT, USA). To optimize the number of informative SNPs, several thresholds for quality control parameters were evaluated. This included removing SNPs having a call rate of less than 0.90, minor allele frequencies (MAF) of less than 0.05, and more than two alleles. In addition, SNPs not mapped to the UMD 3.1 bovine genome assembly [27] or mapped to sex chromosomes were excluded from the analysis. The final genotypes passing these thresholds consisted of 34,748 SNP autosomal markers, with all samples having a sample call rate greater than 0.90, resulting in *n* = 234,164 eligible samples for subsequent analyses.

### 2.3. Principal Component Analysis and Admixture

To define how the parental breeds influence the population structure of the composite breed, we utilized FlashPCA2 [28] for principal component analysis (PCA). The PCA results were then shown graphically using SNP and Variation Suite (SVS) v8.x (Golden Helix, Inc., Bozeman). This strategy was chosen since the maximum number of samples that SVS software can evaluate is limited, whereas FlashPCA2 provides a stable and accurate result for analyzing large genotypic datasets such as ours (*n* = 234,165). Next, the genomic-based breed composition was inferred from the 34,748 SNPs using the ADMIXTURE 1.3.0 program’s maximum likelihood model [29]. The PLINK program version 1.9 [30] was utilized to produce ADMIXTURE data input files. The analysis was conducted with K values ranging from two to four to reflect the genetic background of the breeds under study, beginning with the two-breed cross (Holstein-Friesian (*n* = 58,274) and Jersey (*n* = 35,556)) and stopping with the total number of ancestral populations in the analysis, given the four parental breeds, which included Holstein (*n* = 1786) and Friesian (*n* = 1201) separately as well. As Alexander et al. (2009) described, subsequent cross-validation was employed to determine the most likely value of K. The admixture output was displayed using a customized R v4.2.2 script [31]. To analyze the influence of the number of animals representing each breed in this analysis, a second ADMIXTURE analysis was conducted with a balanced number of individuals by randomly selecting samples to represent each breed (KiwiCross^®^ (*n* = 2045), local Holstein-Friesian (*n* = 1748), Holstein (*n* = 1786), Friesian (*n* = 1201), and Jersey (*n* = 1778)). The outputs of both analyses were used as the basis for the genomic breed composition estimation.

### 2.4. Identification of Genetic Signatures of Selection

**Figure 2:**
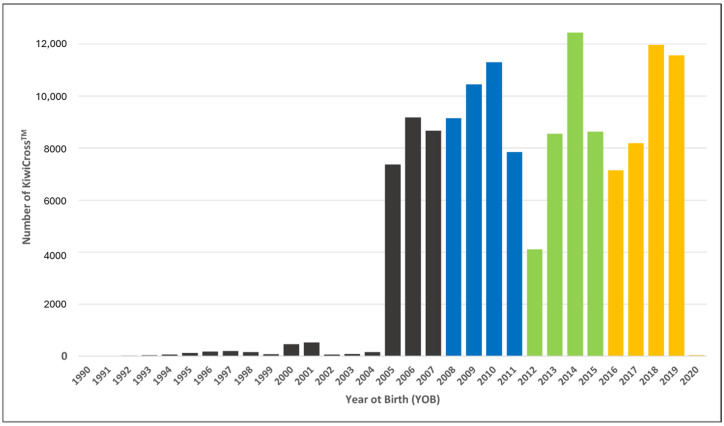
YOB distribution among all 138,774 KiwiCross^®^ colored by year groupings (YOB1, *n* = 27,369, black; YOB2, *n* = 38,751, blue; YOB3, *n* = 33,735, green; and YOB4, *n* = 38,899, yellow).

Animal genotypic data were parsed based on PR performance and only utilized animals which fell into the two extremes of high or low PR. The phenotypic data were initially examined in R version 4.2.2 [31] to determine the data distribution’s normality and opposing tails. The mean value of a characteristic was used for all animals with multiple measurements. Animals within the tail with greater than two standard deviations (SD) were classified as low-performance, whereas those within the tail with less than two SD were classified as high-performance. The second-way data were divided based on year of birth to identify relevant signatures of selection across generations. All KiwiCross^®^ composite cattle were divided into one of four groups based on their year of birth (YOB) with most groups spanning four-year intervals (Figure 2). YOB1 consisted of animals born from 1990 to 2007 (*n* = 27,369), YOB2 included animals born from 2008 to 2012 (*n* = 38,751), YOB3 animals were born from 2013 to 2016 (*n* = 33,735), and the remaining animals born from 2016 to 2020 were in YOB4 (*n* = 38,899). The YOB blocking strategy was to ensure that each group would have a roughly equal representation of animals and capture genetic change or selection over time that a single year or two likely would not show. We note that YOB1 has a range of 18 years as opposed to just four years. However, 93% (*n* = 25,364) of the animals were born in the last four years (2004–2007), allowing us to utilize all animals within the dataset. The signature of selection was then assessed for each category using all 34,748 SNPs. Three statistical approaches focused on recognizing genomic haplotypes were applied to find possible selection signals across performance levels or YOB groups. The first approach aimed to identify haplotype variants from linkage disequilibrium (LD)-degraded patterns between extreme PR performance levels by using a cross-population extended haplotype-based homozygosity score test (XP-EHH) [32]. XP-EHH is a cross-population method for statistically detecting signals of divergent haplotype selection between sub-populations. The second statistical strategy employed hapFLK [33], which includes the hierarchical structure of the population (Reynolds’ genetic distance) [34] to assess haplotype frequencies between different performance levels. This method not only allowed us to compare across many populations simultaneously, unlike pairwise comparison in XP-EHH, but it also allowed us to apply it to unphased genotype data, which were used to validate our phased genotype findings from the previous method. The last statistical analysis used to decode the genetic selection between different performance levels was runs of homozygosity (ROH). ROH, the indicator of genomic autozygosity, may be defined as two contiguous identical-by-descent (IBD) stretches of homozygous haplotypes of a common ancestor in an individual inherited from both of its parents [35,36]. In all, haplotype differentiation between performance levels was identified and assessed in pairwise comparisons of the groups using LD, in a hierarchical structure analysis of haplotype frequency, and through ROH. In addition, haplotype differentiation between YOB groups was also assessed through pairwise comparisons and ROH to support our findings in performance level comparison.

#### 2.4.1. The XP-EHH Pairwise Population Statistic

First, XP-EHH (cross-population EHH) statistic [32] was performed in the *rehh* package [37] in R v. 4.2.2 [31] to identify strong extended homozygosity in *Subpopulation1* relative to *Subpopulation2* and vice versa. To estimate XP-EHH values between two different sub-populations, which in this case were pairwise comparisons between subsequent YOB groups and between high- and low-performance populations, we used the ies2xpehh() function test that computes XP-EHH using two data frames, one for each subpopulation, containing the iES statistics as obtained by the scan_hh() function in the same R package. XP-EHH was constructed to have an approximately standard Gaussian distribution and may be transformed analogously into a *p*-value, which was later plotted using customized R scripts. The Shapeit program [38] was used to phase the genotypes based on their parental source, and the output files from this program were used as input files for the *rehh* package for the analyses. Due to the lack of frequency of genetic recombination information for this crossbreed, we employed a standard physical-to-genetic relationship of 1 Mb to 1 cM throughout the cattle genome for the phasing process, as suggested by the program manual.

#### 2.4.2. hapFLK Test

To examine the genetic selection events between high- and low-performance animals, we used all potential parental sources to run the hapFLK scripts [33]. We chose Ayrshire as the outgroup because it coexisted in New Zealand. We utilized K = 10 and nfit = 30, as recommended by the program manual, given our data structure. We estimated the hapFLK chi-squared density and accompanying *p*-values using Python and R scripts based on the hapFLK results (https://forge-dga.jouy.inra.fr/projects/hapflk/wiki/Options, accessed on 1 June 2023).

#### 2.4.3. ROH Analysis

*DetectRuns* package in R was used to conduct ROH studies for multiple sub-populations of various performance composite breeds [39]. In keeping with the earlier XP-EHH test, ROH analysis will identify ROH islands, which are sections of homozygous sequence in the genome of a substantial proportion of individuals in the population. The parameters were set so that runs could contain up to one heterozygote and five missing genotypes with a maximum gap equal to 100 kb, and the minimum run length was set to 25 kb with a minimum of 25 SNPs. In addition, we also used another filter criteria to identify common ROH within performance levels, including (a) genomic areas that resulted in ROH in >20% of individuals; and (b) comprised more than 10 SNPs to identify the most significant ROH regions. The same R package was also used to calculate the ROH-based inbreeding using the output from the above analysis to calculate individual inbreeding/consanguinity coefficients using the following equation:

$$F_{ROH}=\frac{{\sum L}_{ROH}}{L_{genome}}$$

where ${\sum L}_{ROH}$ is the sum of the length of all ROH detected in an individual, and $L_{genome}$ is the total length of the genome that was used.

### 2.5. Local Ancestry Estimation of Candidate Selection Regions

The efficient local ancestry inference (ELAI) program was used to infer local ancestry in order to comprehend the ancestral origins of the major selection regions in the various composite breed sub-populations [40]. Holstein, Friesian, Jersey, and Holstein-Friesian were the four source populations used for our ELAI analysis. The cluster sizes for the upper and lower layers were set at four and twenty, respectively. In accordance with the ELAI program’s requirement, the number of admixing generations must be specified. So, assuming a three-year generational gap, we defined 10 admixture generations from 1990 to 2020. Similar to the hapFLK test, we conducted 30 independent EM runs with a 20-step length, each before averaging the results across all participants at each locus. The output from ELAI is the estimated ancestral allele dosages for each individual at each SNP. In diploid genomes, such as cattle, each ancestry has the combination of values that sum to two. For example, in two-breed crossbreeding systems, an allele dosage of 1.0 indicates perfect heterozygosity, with each allele originating from each parent. We then used the Student-t test to compare the significance between the average ancestry found in the specific region identified from the previous analysis and the average ancestry across the BTA.

## 3. Results

### 3.1. Principal Component Analysis and Admixture

**Figure 3:**
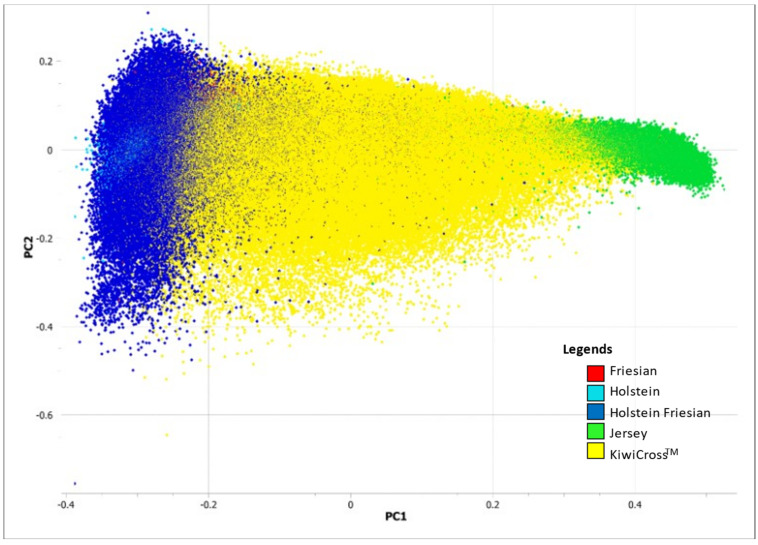
Genomic principal component analysis using 34,748 SNPs to compare the composite breed, KiwiCross^®^ (*n* = 137,348) with their parental breeds, Holstein-Friesian (*n* = 58,274) and Jersey (*n* = 35,556). Purebred Holstein (*n* = 1786) and Friesian (*n* = 1201) populations were also included as the ancestry breeds for Holstein-Friesian in the analysis.

The PCA identified the expected population structure based on breed, with Jersey and Holstein-Friesian showing the main divergence from one another on PC1 (5.65%), and KiwiCross^®^ being intermediate (Figure 3). In general, the KiwiCross^®^ breed consists of three different levels of admixture, which can be seen in the variation in PC1, the first of which is individuals at the center of the PCA diagram that are roughly 50% Holstein-Friesian and 50% Jersey. The other two admixture levels cluster more toward one or the other of the parent breeds. Additionally, our PCA analysis supported our initial expectations of Holstein-Friesian as its own composite breed with variation described by PC2 (0.79%). Although the Holstein-Friesian breed appears to be clustered with its ancestral breeds in one large cluster in Figure 3, subsequent analysis using fewer individuals of more equal proportion representing each breed revealed that the purebred Holstein and Friesian breeds are separate and have their own clusters at opposite ends of the Holstein-Friesian cluster (Supplementary Materials Figure S1). Due to the large number of Holstein-Friesians in the entire dataset, it was difficult to distinguish between purebred Holstein and Friesian and Holstein-Friesian in Figure 2 for which PC2 describes much less variation. However, PC2 in Supplementary Materials Figure S1 more successfully captures the increased variation (11.58%) that distinguished the Holstein-Friesian from the clustered ancestral Holstein and Friesian breeds when using a more balanced dataset of animals representing each population. Overall, both PCA diagrams indicate that Jersey was most distinct from the other populations and more homogeneous, with clear and tight sub-clustering compared to the other breeds.

### 3.2. Genomic Breed Composition Estimation

**Figure 4:**
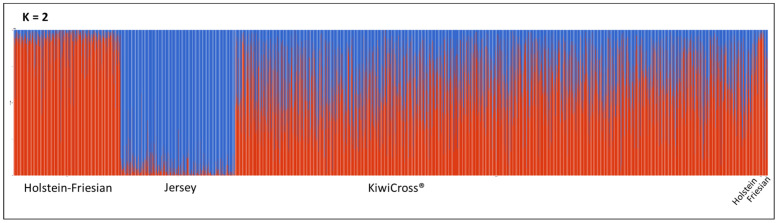
Admixture analysis reflecting optimal genetic clustering at K = 2 for the composite breed, KiwiCross^®^ (n = 137,348), and parental populations of Jersey (n = 35,556) and Holstein-Friesian (n = 58,274). Local Holstein (n = 1786) and Friesian (n = 1201) were also included as ancestral breeds for Holstein-Friesian. However, due to having significantly fewer individuals representing both breeds, the breed composition was not distinctive. Individual vertical bars along the X-axis represent individual cattle grouped by breed; the Y-axis provides a measure of the population composition of each genetic breed found within individuals using 34,748 SNPs.

To fully understand the genomic breed composition of the KiwiCross^®^, an unsupervised clustering method using the software ADMIXTURE was used with K = 2 to K = 4 based on the expected parental source in the dataset. The cross-validation (CV) error was lowest at K = 2 (0.30445) (Figure 4), suggesting the optimal population differentiation is distinguishing the main ancestry sources of Holstein-Friesian and Jersey. KiwiCross^®^ average breed composition in this ADMIXTURE analysis was 43.3% Jersey and 56.7% Holstein-Friesian. As expected, the ancestral Holstein had higher similarity with Holstein-Friesian at 94.8%, while Friesian was more admixed, with an average of 32.2% Jersey’s genetic signature. However, due to the significantly higher number of animals representing the Jersey and Holstein-Friesian populations, results may be skewed by these populations. Additionally, the KiwiCross^®^ population predominates the cluster overlap, similar to our principal component analysis, causing the Holstein population to fall under the same cluster as the Holstein-Friesian population while the Friesian population clustered together with the KiwiCross^®^ population.

**Figure 5:**
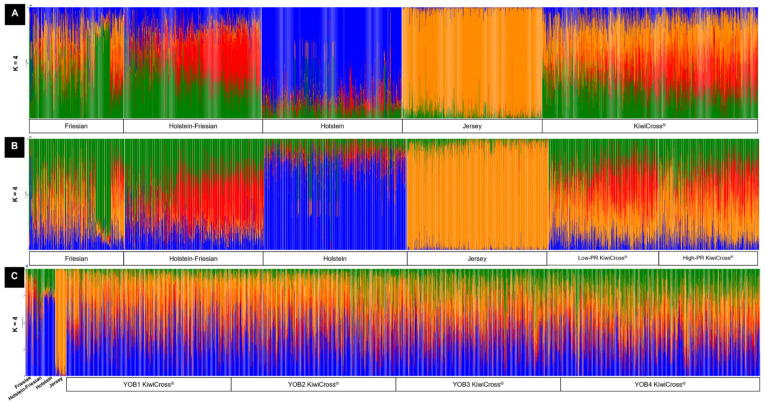
Admixture analysis reflecting genetic clustering of breeds using (**A**) the balanced number of composite breeds, KiwiCross^®^ (*n* = 2678); (**B**) low-performance KiwiCross^®^ (*n* = 1376) and high-performance KiwiCross^®^ (*n* = 1259; and (**C**) different YOB groups (YOB1, *n* = 27,369; YOB2, *n* = 38,751; YOB3, *n* = 33,735; and YOB4, *n* = 38,899). All three admixtures were run using a balanced parental population of Jersey (*n* = 1778), Holstein-Friesian (*n* = 1748), Friesian (*n* = 1201), and Holstein (*n* = 1786), for K = 4. Individual vertical bars along the X-axis represent individual cattle grouped by breed; the Y-axis provides a measure of the population composition of each genetic breed found within individuals using 34,748 SNPs.

A more balanced dataset of roughly equal numbers of animals representing each population was assessed to identify whether the larger populations were skewing the interpretation of admixture results (Figure 5A). This ADMIXTURE analysis continued to exhibit the same homogenous trend for Jersey, but also showed distinct breed signature patterns for Holstein-Friesian and both purebred Holstein and Friesian. Contrary to using the whole dataset, this analysis’ lowest CV error (0.54972) revealed that four clusters best describe the admixture across the five populations. The composite breed KiwiCross^®^ contained a combination of breed ancestry from its parental breeds, with the average KiwiCross^®^ having a genomic breed composition of 39.8% Jersey, 26.8% Friesian, 9% Holstein, and 24.4% Holstein-Friesian (Figure 5A). Using the four ancestral breeds, we see a significant decrease in the estimate of the Holstein-Friesian breed from 56.7% to 24.4%, yet a total Holstein-Friesian, Holstein, and Friesian composition that increased by 3.5% from the two-breed estimation to 60.2%.

Thus, to ensure that we captured the unique genetic signature of all the potential parental sources in the dataset, we used the balanced parental source dataset to further explore the relationship between global estimated breed composition between KiwiCross^®^ performance levels (Figure 5B) and YOB groups (Figure 5C). Using the same balanced parental populations, first we conducted ADMIXTURE to determine the differences in breed composition by performance levels. We then used all KiwiCross^®^ but grouped them according to the YOB categories. Since the pedigree-based breed estimations provided only differentiated the three breeds of Jersey, Holstein-Friesian, and Friesian, we combined the genomic estimates of Holstein and Holstein-Friesian as a single estimate for comparison purposes. Our global genomic-based ancestral breed composition revealed a significant difference (*p* < 0.05) between both performance and YOB groups (Table 1) for Holstein-Friesian and Jersey composition. Higher Holstein-Friesian composition was present in the most recent newborn animals (YOB4) (43.5%), suggesting recent KiwiCross^®^ selection may favor the Holstein-Friesian breed compared to Jersey. However, because of the KiwiCross^®^ open crossbreeding approach that includes mating to purebred or crossbreed lineages as desired, this pattern may be the product of recent breeding with Holstein-Friesian or Holstein, or an additive accumulation of Holstein-Friesian or Holstein lineage within KiwiCross^®^ mated to one another. Comparison of PR performance levels showed a significant increase in Jersey lineage (36.8% to 42.5%) in high-PR-performance KiwiCross^®^ countered by a significant decrease in Holstein-Friesian composition from 36.4% to 30.7% in high-performance animals. There was no significant impact in the global estimate of Friesian for either category. Lastly, as expected, there was a significant difference between pedigree and genomic-based breed estimates, except for Jersey composition estimate in several groups. This may be due to the fact that Jersey has more homogenous populations compared to the other ancestral breeds, as observed in admixture results (Figure 5). The genomic breed composition produced from the Q matrix in unsupervised mode offers more robust result estimations [41], as the reliability of pedigree-estimated breed composition can be compromised by missing, inaccurate, or incomplete records [42].

### 3.3. Identification of Genetic Signatures

**Figure 6:**
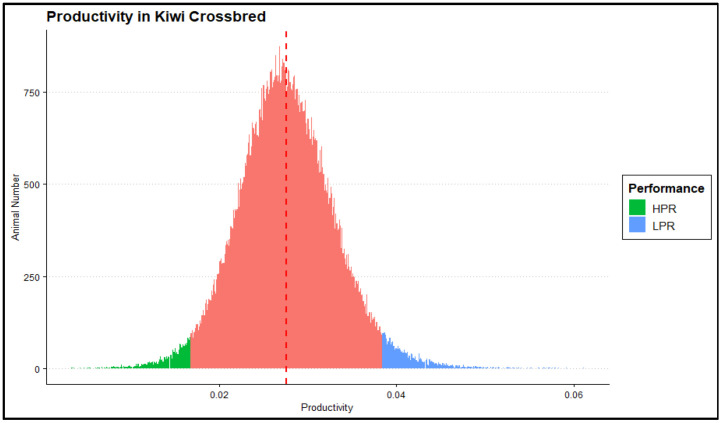
Distribution of productivity performance (PR) measurements and identification of extreme performance levels within tails of the distribution. Colored by different performance categories, (blue—low-performance (LPR), *n* = 1376; green—high-performance (HPR), *n* = 1259; red—average performance, *n* = 134,713). The dotted vertical red line denoted the mean measurement of the trait (mean: 0.028 ± 5.43 × 10^−6^).

For KiwiCross^®^ composite breeding, generating animals that increase milk production while maintaining constant or lower live weight is desirable. The further the animal’s score from a zero value in the PR trait distribution, the lower productivity performance. Following this criterion and using two SD from the mean as a cutoff, we classified 1376 animals with the highest PR values (furthest from 0) as low-performance (mean: 0.044 ± 8.75 × 10^−6^) and 1259 animals with scores closest to zero, or lowest PR values, as high-performance (mean: 0.013 ± 4.23 × 10^−6^) (Figure 6). Results from the various selection methods were then compared to identify common regions across analyses as a way to prioritize the potential importance of the regions identified for the performance levels.

#### 3.3.1. Haplotype Differentiation Based on XP-EHH

**Figure 7:**
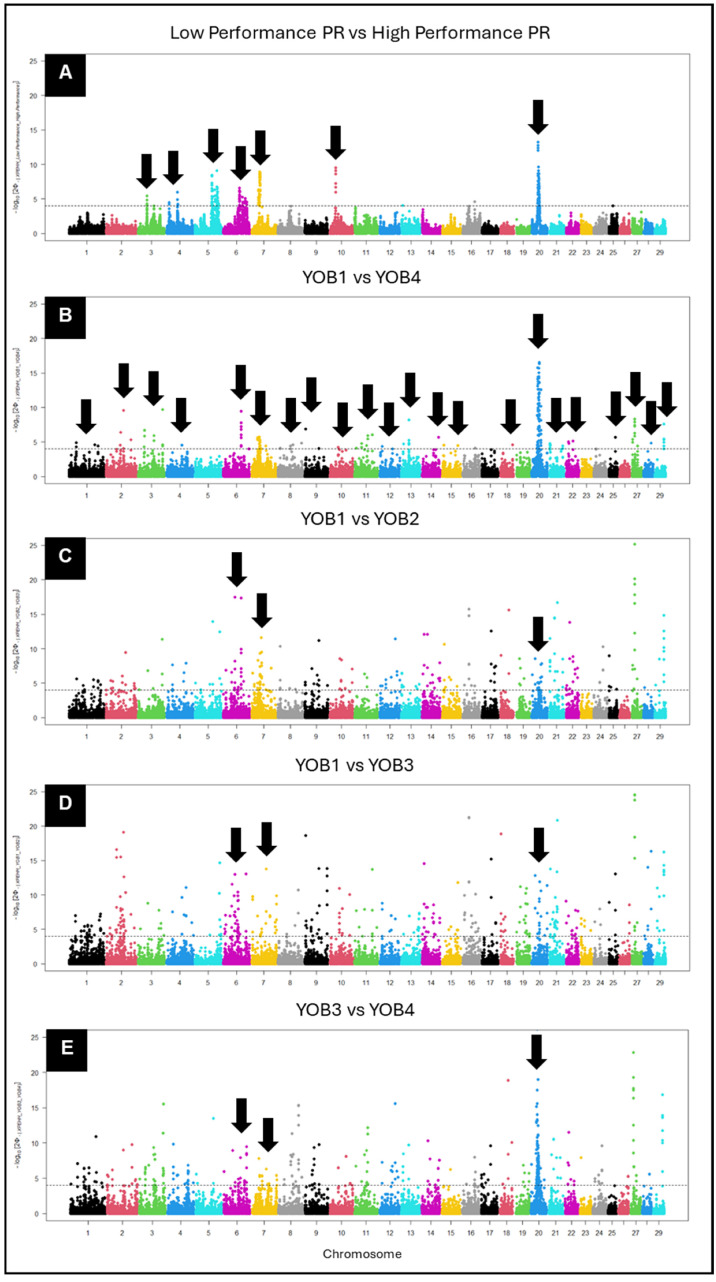
Manhattan plot of a cross-population extended haplotype homozygosity (XP-EHH) score for pairwise comparisons between (**A**) low-performance vs. high-Performance, (**B**) YOB1 vs. YOB4, (**C**) YOB1 vs. YOB2, (**D**) YOB2 vs. YOB3, and (**E**) YOB3 vs. YOB4. The significance threshold depicted by the horizontal line was set at 4 as suggested by the *rehh* package manual. The downward arrows identify genomic regions where there was significant population differentiation (*p*-value < 0.5). The dots within the plot represent single-nucleotide polymorphisms (SNPs) respective of their genomic location. The color of the dot (SNP) represents the chromosome it is located on with each color change reflecting a change in chromosome.

First, we used the XP-EHH approach to perform pairwise comparisons of haplotype variation between low- and high-performing KiwiCross^®^ cattle and between the YOB groups (YOB1 vs. YOB2, YOB2 vs. YOB3, YOB3 vs. YOB4, and YOB1 vs. YOB4) (Figure 7). We observed a clear signal of haplotype differentiation between low- and high-PR-performance animals on BTA 3, 4, 5, 6, 7, 10, and 20 (Figure 7A). Comparing the earliest YOB1 group and the most recent KiwiCross^®^ YOB4 showed the greatest selection divergence in regions on BTA 1, 2, 3, 4, 6, 7, 8, 9, 10, 11, 12, 13, 14, 15, 18, 20, 21, 22, 25, 27, 28, and 29 (Figure 7B). Comparison of the performance levels and YOB groups identified the same signatures of selection, or genomic regions, on BTA 6, 7, and 20 (Supplementary Materials Figure S2). Tracking these three regions across the YOB groups showed the degree of differentiation of all positions changing over time. On BTA 20, association became stronger over time exemplified by the increasing -log10*p*-values of the haplotype differentiation from YOB1 to YOB4. In contrast to the regions found on BTA 20, the signatures on BTA 6 and 7 decreased over time.

#### 3.3.2. Selection Signatures Based on hapFLK

**Figure 8:**
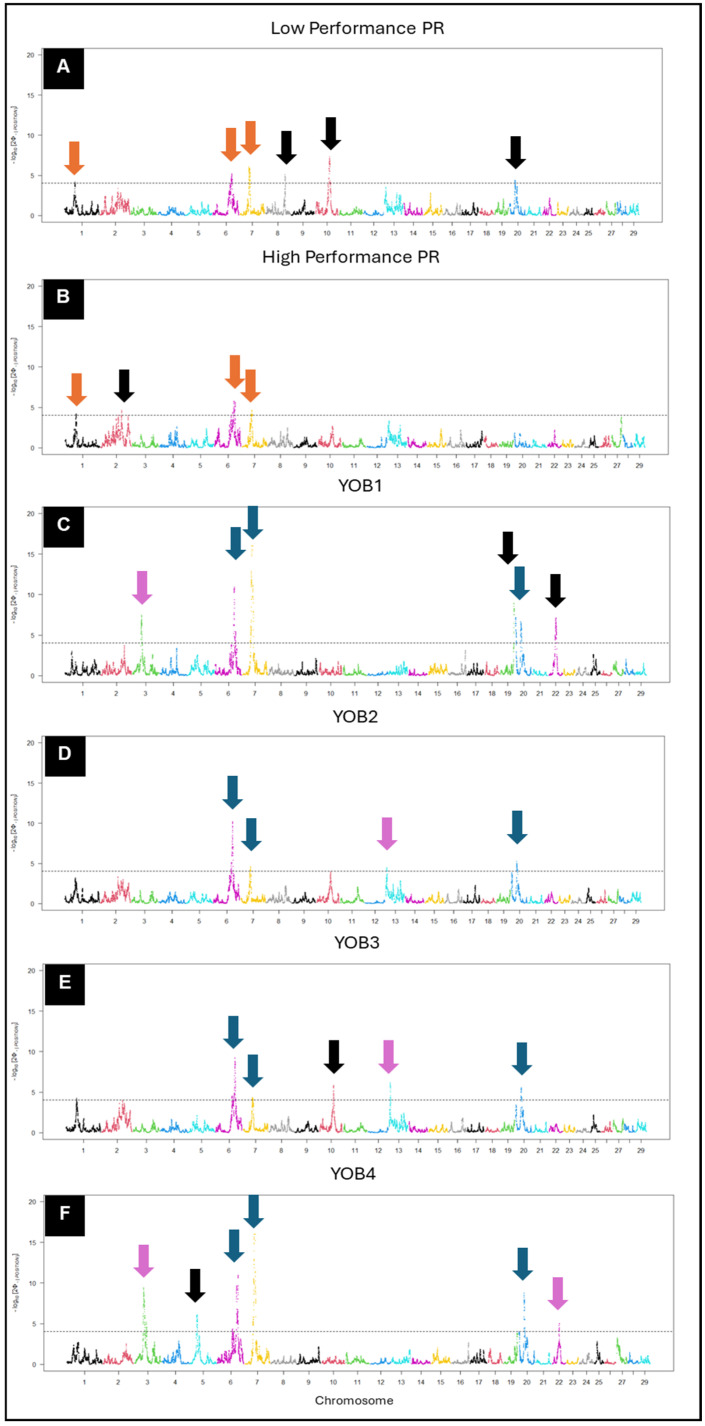
Manhattan plot of hapFLK score for (**A**) low-performance PR (n = 1376); (**B**) high-performance PR (n = 1259); (**C**) YOB1 (n = 27,369); (**D**) YOB2 (n = 38,751); (**E**) YOB3 (n = 33,735); and (**F**) YOB4 (n = 38,899). Each group was analyzed against all four parental sources with Ayrshire as an outgroup. The significance threshold depicted by the horizontal line was set at 4 for uniform purposes with the XP-EHH method. The downward arrows denote regions either common across categories (high- and low-performance = orange; across all YOB groups = blue; and across at least two YOB groups = light blue) or unique within a specific category (black). The dots within the plot represent single-nucleotide polymorphisms (SNPs) respective of their genomic location. The color of the dot (SNP) represents the chromosome it is located on with each color change reflecting a change in chromosome.

Under this approach, we observed two types of signals detected: ones common across categories and ones uniquely detected within certain categories (Figure 8). First, we discovered significant signals common to both PR performance levels on BTA 1, 6, and 7, with additional unique signals on BTA 8, 10, and 20 in low-performance and BTA 2 in high-performance animals (Figure 8A,B). Looking across the YOB groups, we observed the same repeating signals of selection on BTA 6, 7, and 20 that exist in all four YOB groups. Other signals were uniquely detected in certain YOB groups. For example, significant selection signatures on BTA 3 and 22 are detected in YOB1 and YOB4 (Figure 8C,F), while signatures of selection on BTA 19, 10, and 5 are only apparent in YOB1, YOB3, and YOB4 (Figure 8C,E,F), respectively. Another notable observation in comparing the signatures of selection across YOB groups is the degree of association for the regions of selection. Signals on BTA 7 and 20 were more highly associated in earlier generations of KiwiCross^®^ but then decreased before once again increasing in the modern born KiwiCross^®^ (YOB4). Supplementary Materials Figure S3 shows association plots of the significant signals detected on BTA 6, 7, and 20 across multiple groups. Generally compared to the XP-EHH *p*-values, hapFLK produced lower *p*-values potentially due to the hierarchical structure of the population being included in the algorithms that help in reducing false positive signals within the studied categories.

#### 3.3.3. Selection Signatures Based on ROH

**Figure 9:**
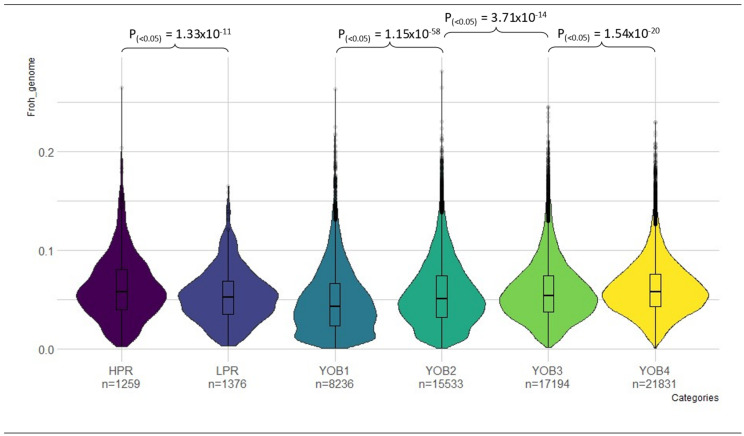
Significant differences (*p* < 0.05) in inbreeding-based ROH (FROH) were found when comparing between high–low performance levels and between YOB groups. Varying colors distinguish the different groups: HPR—purple, LPR—violet, YOB1—teal, YOB2—green, YOB3—light green, YOB4—yellow.

Table 2 lists the proportion of ROH by size within the PR performance levels and YOB groups. In general, within the YOB groups, the modern-born KiwiCross^®^ identified the most ROHs in the shorter-length category (5 Mb) and longer-length category (>40 Mb). Although modern-born animals’ inbreeding-based ROH (0.0616) is comparatively low, we can identify a substantial rise in inbreeding (*p*-value < 0.05) between the YOB groups from the most historical to the most recent YOB (Figure 9). Interestingly, the inbreeding value greatly varies between the performance levels, with the high-performance having higher inbreeding (Figure 9), which is also reflected by the proportion of ROH regions detected in high-performance animals. However, as both performance levels included animals born in every YOB group, this cannot be directly attributed to YOB. Seven ROH regions were found on BTA 1, 5, 6, 7, and 16 in high-performance animals and another seven regions were on BTA 1, 5, 7, and 16 and 20 in low-performance animals. In contrast, 11 regions were found spanning four BTA (BTA 1, 5, 16, and 20) in the YOB groups. These included one ROH on BTA 5 for YOB1 animals, two ROH on BTA 1 and 5 for YOB2 animals, three ROH on BTA 1, 5, and sixteen for YOB3 animals, and five ROH regions on BTA 1, 5, 6, and 20 for YOB4 animals.

### 3.4. Identification of the Overlapping Signatures of Selection from Multiple Analyses

**Figure 10:**
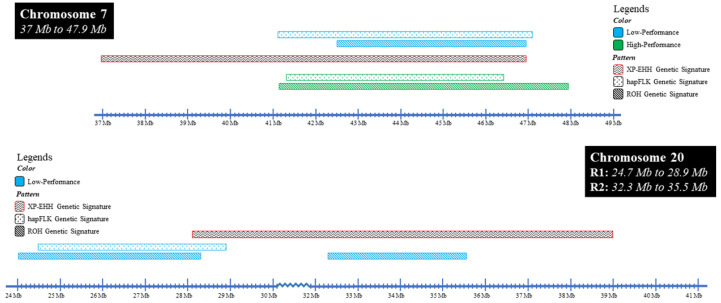
Location of overlapping regions (BTA 7 and BTA 20) detected in XP-EHH, HapFLK, and ROH between high–low productivity performance levels. The region on BTA 7 was found in both high- and low-PR animals whereas two regions were identified in only the low-performance group on BTA 20. Colors and shading of horizontal bars show where specific analyses identified a signal of selection.

To understand which selection signatures are most important in productivity performance, we first evaluated the regions commonly identified in the high- or low-performance levels in all three approaches (XP-EHH, HapFLK, and ROH) (Table 3). Of all the regions detected across the genome, only two BTA yielded strong signals from multiple analyses. For the high-performance animals, only the region on BTA 7 ranging from 41.2 Mb to 46.9 Mb was identified by all three methods (Figure 10). Despite haplotype differentiation detected through the XP-EHH method (Supplementary Materials Figure S2) on BTA 20 between high and low performance, the signal produced from the other two analysis (hapFLK and ROH) was not significant in the high-performance animals. In the low-performance group, three regions were found, including the same region on BTA 7 between 41.2 Mb and 46.9 Mb and two regions on BTA 20 between 24.1 Mb to 28.3 Mb and 32.3 to 35.6 Mb (Figure 10). We further analyzed all these target regions by using the plot_SnpsInRuns function from the R package *detectRUNS* to plot the number of times each SNP was found within a ROH run. This plot revealed a pattern of increasing numbers of animals having the same ROH regions both in BTA 7 and 20 over time (i.e., YOB category). This could indicate that with increasing selection over time, there is some correlation between these three regions and performance (Supplementary Materials Figure S4).

### 3.5. Local Ancestry Estimation of Selection Regions

**Figure 11:**
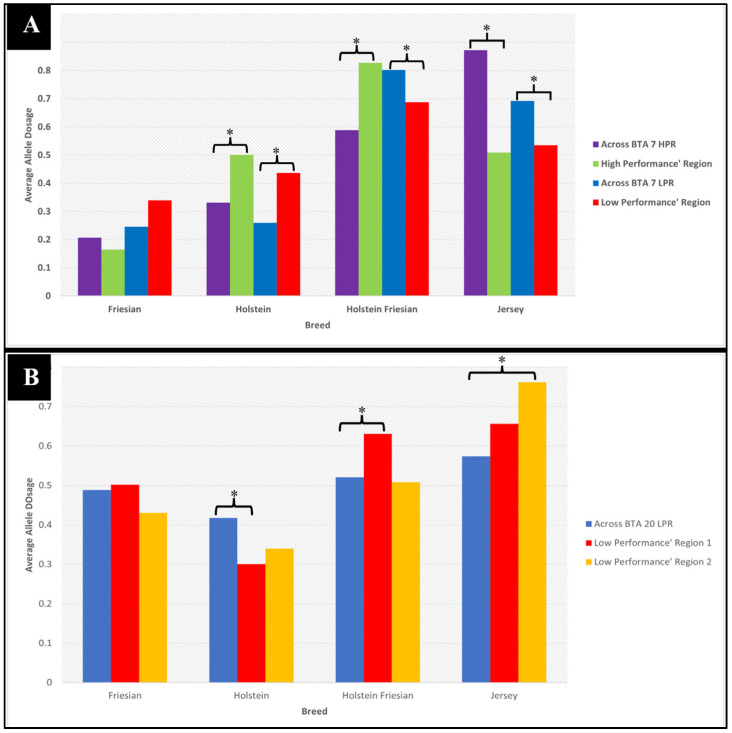
Comparison of the average allele dosage of ancestry breeds within KiwiCross^®^ on (**A**) BTA 7 (targeted region: 37 Mb to 47.9 Mb) and (**B**) BTA 20 (targeted region 1: 24.7 Mb to 28.3 Mb, region 2: 33 Mb to 35.5 Mb). * denotes a significant difference with *p*-value < 0.05.

To identify which ancestry was associated with the regions of selection, we calculated the mean for each ancestry breed using the estimated ancestral allele doses produced for each individual at each locus for each high- or low-performance level (Figure 11). The ancestry breed with the highest average allele dosage was designated as the ancestral origin for an SNP within a certain performance level. The first target region on BTA 7 (37 Mb to 47.9 Mb) in the high-performance animals have higher average Holstein and Holstein-Friesian allele dosage (0.50 and 0.83, respectively) from their average allele dosage across the entirety of BTA 7 (0.33 and 0.59, respectively). In contrast, the average Jersey allele dosage across the entirety of BTA 7 is 0.87, but in this target region, the average Jersey allele dosage is 0.51, which is significantly lower than the average across the chromosome (*p* < 0.05). There was no significant difference between Friesian allele dosage across BTA 7 and the targeted region for Friesian ancestry in the high-performance animals. With this observation, we can infer that the higher Holstein and Holstein-Friesian ancestry in this region may be associated with the high-performance level. Evaluation of the same targeted region, but in the low-productivity group of animals, showed almost the same observation as in the high-performance group, with a significantly lower Jersey allele dosage average (0.54) and higher Holstein allele dosage average (0.44) compared to the average allele dosage across BTA 7 for Jersey (0.69) and Holstein (0.26). However, compared to the high-performance group animals, the low-performance group had a lower Holstein-Friesian allele dosage (0.69) compared to their average (0.80) in this region. However, it was still the greatest when compared to the other three breeds. Further PCA analysis, not shown here, focusing only on SNPs within these regions, showed that sub-clustering of these two performance levels suggests that a different variation exists between them despite the common Holstein-Friesian ancestry (Supplementary Materials Figure S5). The first targeted region on BTA 20 (24.7 Mb to 28.9) for the low-performance group showed the same allele dosage pattern as in BTA 7, with Jersey having the lower (0.66) and Holstein-Friesian the higher (0.63) allele dosage compared to their BTA 20 average (0.57 and 0.52, respectively). Although Jersey has the highest ancestry compared to Holstein-Friesian in this region, no significant difference was found when comparing Jersey ancestry across BTA 20. On the other hand, Holstein-Friesian ancestry is significantly increased in this region compared to the average in BTA 20, which could indicate a stronger association of this ancestry with low performance levels at this genomic region. Interestingly, the second targeted region on BTA 20 (32.3 Mb to 35.5 Mb) for the low-performance group showed significantly higher allele dosage in Jersey (0.76) compared to allele dosage in Holstein-Friesian (0.51) and Holstein (0.33). No significant difference was observed for the Friesian breed composition when we compared the average within both targeted regions and across BTA 20.

## 4. Discussion

Findings from this study relating ancestry to important regions of production selection demonstrate the complex genomic architecture created in the KiwiCross^®^ over several generations of development. The PCA analysis revealed the expected relationship between the composite breed, KiwiCross^®^, ranging between the two parent breeds, Holstein-Friesian, and Jersey (Figure 3). Similarly, the PCA using a balanced dataset of animals per population identified this same trend for the Holstein-Friesian, originally a composite breed, compared to Holstein and Friesian populations on PC2 (Supplementary Materials Figure S1). Notably, the purebred Friesians had a higher overlap with Holstein-Friesians than Holstein. Historically, the Holstein-Friesian in New Zealand descended from animals imported from the west coast of the United States in 1925. They had a closed breeding system until a significant influx of North American Holstein cattle imports were received from Canada in the 1960s and 1970s. This was followed in the 1980s by the importation of US genetics and, more recently, by the importation of European genetics, primarily Dutch Holstein [17]. Thus, it is plausible that New Zealand Holstein-Friesians and Friesians will be more comparable to purebred Holsteins due to the origin of purebred Friesians, which originated in Friesland or what is now the Netherlands [6,7]. On the other hand, due to this historical record also, a remnant of the 1960s’ closed Holstein-Friesian breeding system may be found in the cluster of Holsteins, which can explain a clear, unique genetic admixture signal that was not clustering with other breeds used in this study. In addition, as Holstein-Friesian cattle have been continuously selected over the past 2000 years as a composite breed, they developed their own distinct genetic signature indicated by our admixture results differentiating Holstein-Friesian individuals, as shown in Figure 5A, that may have originated from the North American Holstein-Friesian genetics, which carried traits for high milk yield per cow [17].

Examination of the KiwiCross^®^ revealed the different levels of relationship and breed composition among individuals in both PCA and admixture analysis. This indicates how historical breeding created KiwiCross^®^ with varying degrees of admixture, which may influence performance. The unique chromosomal segment combinations produced when breeding KiwiCross^®^ may impact the genetic diversity affecting heterosis or the inheritance of distinct advantageous parental breed characteristics. A benefit of crossbreeding is that heterosis and inbreeding depression are negatively correlated, i.e., when one increases, the other decreases. Both pedigree and ROH inbreeding levels (Figure 9) increased from YOB1 to YOB4. Although the estimated value is considered low by inbreeding scales, inbreeding is increasing between each generation category, with a relatively constant increase of 0.2% in the last two generation categories (0.4% total in 8 years), with modern-born KiwiCross^®^ (born in 2016 and after) having the greatest inbreeding value of 0.0616 FROH. Animals with superior productivity had higher levels of inbreeding (0.0623), similar to the average of the modern generation category, than animals with lower productivity levels, suggesting increased selection within the high-performance group of specific family lineages. Interestingly, when we separated the animals in the performance groups into YOB groups and recalculated the average of both inbreeding estimations, the results show that the average inbreeding for the high-performance group was nearly the same across all generations (min: 0.060, max: 0.063); however, the average inbreeding within the low-performance group increases in each subsequent generation (min: 0.042, max: 0.060). This suggests a more constant level of inbreeding among high-performance animals, whereas the change in inbreeding among the low-performance animals may reflect the general increase in inbreeding of the total KiwiCross^®^ population over time.

Four overlapping regions on two BTA, 7 and 20, were identified from three genetic signature algorithms showing selection within the KiwiCross^®^. One selected region was found in high-productivity animals on BTA 7, which showed higher Holstein-Friesian and Holstein ancestry. This same region was significantly associated with low-productivity animals in which Holstein ancestry was again predominant, yet PCA of the SNPs within this region differentiated the high- and low-performing animals. This suggests that differing ancestral haplotypes are found at higher frequencies in the two performance groups at the same region, and the inheritance of one Holstein or a Holstein-Friesian-derived haplotype is advantageous for the high-productivity animals, while the inheritance of a different Holstein derived haplotype negatively impacts the low-performance group. Jersey ancestry was significantly decreased in both of the performance groups in this region, supporting the concept that it is the Holstien derived haplotypes in this region that are the most impactful. BTA 20 contained two regions of selection relatively close together that were associated with low productivity. Inheritance of a Holstein-Friesian haplotype in the first region and inheritance of a Jersey-derived haplotype in the second region were associated with low-performance animals. Despite the significant increase in Holstein-Friesian ancestry at the first region, haplotypes representing just Holstein ancestry significantly decreased in this region in low-performance animals.

One hundred and seventy-six genes were found across both performance groups in the four performance selected regions. However, only nine genes on BTA 7 were previously annotated as functionally important to Holstein based on the CattleQTLdb (Release 49), including *ABCA7*, *CLK4*, *GDF9*, *MADCAM1*, *NLRP3*, *PROP1*, *RMND5B*, *SLC25A48*, and *SLC34A1* [43,44,45,46,47,48,49,50]. These genes were reported to influence three trait types, including exterior or conformation (five genes—*CLK4*, *MADCAM1*, *NLRP3*, *RMND5B*, and *SLC34A1*), performance (milk/production) (three genes—*ABCA7*, *PROP1*, and *RMND5B*), and reproduction traits (four genes—*GDF9*, *PROP1*, *RMND5B*, and *SLC25A48*). In addition, *PROP1* was also reported to play an important role in exterior traits in other breeds, such as Qinchuan, Luxi, Nanyang, and Jiaxian Red breeds [43]. Our findings suggest that the advantageous variant of *PROP1* may also be favored for selection in Holstein-Friesian cattle, given its location within our selection region and its association with ancestral inheritance in high-performance animals. The differentiation and survival of anterior pituitary cells, which create hormones that control metabolism, growth, reproduction, and other physiological activities, depend critically on *PROP1*. The secretion of numerous pituitary hormones is impacted by combined pituitary hormone deficit (CPHD) caused by a loss-of-function mutation in the *PROP1* gene in humans and mice [51]. Numerous symptoms, including dwarfism, infertility, and decreased milk production, are brought on by CPHD. Another crucial gene in this area is *RMND5B*, a member of the endoplasmic reticulum (ER) stress response gene family. Because it encodes a chaperone protein that aids in the folding and appropriate operating of other ER proteins, *RMND5B* is an essential part of the unfolded protein response (UPR). Variations in the *RMND5B* gene have been linked to various attributes in cattle, including feed efficiency, carcass quality, and muscle growth [52]. Our findings of positive and negative Holstein-derived haplotypes on BTA 7 support known characteristics of the Holstein breed for superior production and increased stature, yet historic reproduction deficiencies. It is plausible that the advantageous derived haplotypes from Holstein or Holstein-Friesian in this region relate to superior production while the negative haplotypes from Holstein relate to exceptionally large stature (and/or poor reproduction), reducing productive efficiency.

On BTA 20, specifically for low performance animals, nine genes were found in the first region and 13 genes in the second region. Of these 22 genes, only 12 genes were annotated based on the CattleQTLdb (Release 49), of which 11 were reported to be functionally important in the Holstein breed, including *C6*, *C7*, *DAB2*, *FST*, *ITGA1*, *NDUFD4*, *PARP8*, *PELO*, *PLCXD3*, *RICTOR*, and *TTC33* [44,53,54,55,56,57]. All eleven genes were associated with milk performance traits, with only the *FST* gene also reported for affecting reproduction traits. Interestingly, previous studies also demonstrated strong evidence of three other genes within this identified region (*ARL15*, *FST*, and *RICTOR*) associated with meat traits in Holstein, Charolais, Limousine, and Hanwoo [44,53,55]. Six genes, of which five were associated with milk performance (*FST*, *ITGA1*, *NDUFS4*, *PARP8*, and *PELO*) and two were associated with exterior and reproduction traits (*ARL15*, *FST*), were within the first region of BTA 20. We hypothesize that this group of low-performance animals is inheriting an unfavorable haplotype from Holstein-Friesian and show a significant reduction in Holstein ancestry at this region. Another six genes in the second region of BTA 20 have been associated with Holstein milk production (*C6*, *C7*, *DAB2*, *PLCXD3*, *RICTOR*, and *TTC33*). The low-performing KiwiCross^®^ were found to have a higher prevalence of Jersey-derived haplotypes in this region, which may make them less competitive in overall milk production compared to their high-performing counterparts. However, further investigation into this region also found *RICTOR*, *PLCXD3*, and *TTC33* genes implicated in regulating growth and development [58,59], which may play a role in the selection of body size in the KiwiCross^®^ as part of their production efficiency.

The findings of this study support the notion that performance in composite breeds is influenced by allele heritage from parental breeds in addition to the transmission of specific gene mutations. Although the Holstein-Friesian ancestry bias will be offset by the selection index of the PR trait employed, there is still undeniable proof of severe selection on exterior qualities, as shown by the list of genes expressed in the low-performance group. It was also intriguing that some regions showed specific increases and decreases of differing ancestries, such as between Holstein-Friesian, Holstein, and Jersey, whereas the same region on BTA 7 was important for both high and low PR groups, with Holstein-derived haplotypes being both beneficial or detrimental. This observation could also potentially indicate heterosis depression, in which certain genes are rendered inactive due to haplotype breakdown from recombination. Based on our findings, ancestral-derived haplotypes are being selected for or against, which may impact diversity in subsequent KiwiCross^®^ generations. Lastly, the findings of this study illustrate how selection factors can influence a composite breed’s genetic makeup, supporting the notion that a composite breed’s genetic makeup is dynamic and shifts over generations, with key regions of selection being maintained or increasing/decreasing based on selection pressures [13].

## 5. Conclusions

Our results highlight different regions of selection over generations and within KiwiCross^®^ performance groups and link this selection to ancestry. We demonstrated that different lineages or ancestry-specific haplotypes were overrepresented in the chromosomal regions of selection, which differentially affected the genome architecture of the composite breed and the productivity level of KiwiCross^®^. Therefore, breeders of composite breeds need to be aware that any selection for specific traits that favor one of the ancestral breeds over the other can and will alter the proportions of the parental breeds within the crossbred. This may be beneficial for the preservation and expression of desired breed-specific characteristics, but may also diminish the heterosis benefits or result in the loss of rare alleles and diversity in the crossbred population. Specifically, the ancestry-specific haplotypes identified in this project for KiwiCross^®^ can be incorporated into breeding strategies to improve production efficiency. Individuals with more desirable ancestry-specific haplotypes would have a greater chance of contributing to the next generation. However, because desired haplotypes may come from different ancestral sources, selection progress may be slower, similar to negatively correlated traits, and rely on recombination breaking up larger, linked haplotype blocks derived from the same ancestor breed. As the composite breed evolves due to selection for improved production efficiency, breeding strategies used within purebred populations to reduce loss of genetic diversity are required. Currently, within the KiwiCross^®^ population, the generation of future offspring balances selection for improved production efficiency with minimizing the genomic and pedigree co-ancestry among mates, both within the breeding program and on-farm.

## Figures and Tables

**Table 1:** Genomic-based breed composition and pedigree-based breed composition at K = 4 between high–low productivity performance levels and different YOB groups.

Breed	Productivity Efficiency (PR)		Year of Birth (YOB)			
	Low-Performance (*n* = 1376)	High-Performance (*n* = 1259)	YOB1 (*n* = 27,369)	YOB2 (*n* = 38,751)	YOB3 (*n* = 33,735)	YOB4 (*n* = 38,899)
Genomic Holstein-Friesian (Holstein Friesian + Holstein)	0.3642 ± 0.0293 *^#^	0.3067 ± 0.0296 *^#^	0.3724 ± 0.0543 *^#^	0.3437 ± 0.0537 *^#^	0.3892 ± 0.0515 *^#^	0.4348 ± 0.0319 *^#^
Pedigree Holstein-Friesian	0.3230 ± 0.0186 *^#^	0.2752 ± 0.0209 *^#^	0.2751 ± 0.0355 ^#^	0.2793 ± 0.0276 *^#^	0.3296 ± 0.0217 *^#^	0.3655 ± 0.0184 *^#^
Genomic Friesian	0.2675 ± 0.0165 ^#^	0.2891 ± 0.0136 ^#^	0.2515 ± 0.0194 ^#^	0.2687 ± 0.0190 ^#^	0.2783 ± 0.0181 ^#^	0.2647 ± 0.0109 ^#^
Pedigree Friesian	0.2905 ± 0.0108 ^#^	0.3067 ± 0.0296 ^#^	0.3396 ± 0.0221 *^#^	0.3079 ± 0.0165 *^#^	0.2991 ± 0.0113 ^#^	0.2933 ± 0.0097 ^#^
Genomic Jersey	0.3683 ± 0.0331 *^#^	0.4249 ± 0.0426 *	0.3761 ± 0.0559	0.3876 ± 0.0543 *	0.3325 ± 0.0436 *^#^	0.3005 ± 0.0413 *^#^
Pedigree Jersey	0.3817 ± 0.0306 *^#^	0.4317 ± 0.0379 *	0.3830 ± 0.0562 *	0.4091 ± 0.0606 *	0.3659 ± 0.0396 *^#^	0.3354 ± 0.0357 *^#^

* symbol denotes significant differences found (*p* < 0.05) between high- and low-productivity performance groups and across YOB groups. # symbol denotes significant differences found (*p* < 0.05) between genomic-based breed composition and pedigree-based breeds for high- and low-productivity performance levels and across YOB groups.

**Table 2:** Variation in the proportion of ROH by length identified in each performance level and YOB group corrected by the number of animals in each group.

ROH Length	Productivity Efficiency (PR)		Year of Birth (YOB)			
	Low-Performance (n = 1376)	High-Performance (n = 1259)	YOB1 (n = 27,369)	YOB2 (n = 38,751)	YOB3 (n = 33,735)	YOB4 (n = 38,899)
0–5 Mb	25.03	26.67	7.17	10.53	13.43	14.64
5–10 Mb	4.89	5.05	1.24	1.92	2.54	2.96
10–20 Mb	1.20	2.10	0.46	0.72	1.01	1.23
20–40 Mb	0.57	0.58	0.13	0.21	0.30	0.37
>40 Mb	0.09	0.08	0.02	0.03	0.04	0.05

**Table 3:** Genomic regions with overlapping signatures of selection for production efficiency.

PR Group	Ancestry Breed ^1^	Chromosome	Position (Mb)	Annotated Gene and Associated Trait ^2^
High	↑Holstein, ↑Holstein-Friesian, ↓Jersey	7	41.2–46.9	Exterior/conformation: *CLK4*, *MADCAM1*, *NLRP3*, *RMND5B*, *SLC34A1*, and *PROP1* Milk production: *ABCA7*, *PROP1*, and *RMND5B* Reproduction: *GDF9*, *PROP1*, *RMND5B*, and *SLC25A48*
Low	↑Holstein, ↓Holstein-Friesian, ↓Jersey			
Low	↑Holstein-Friesian, ↓Holstein	20	24.1–28.3	Exterior/conformation: *ARL15*, *FST* Milk production: *FST*, *ITGA1*, *NDUFS4*, *PARP8*, and *PELO* Reproduction: *ARL15*, *FST* Meat production: *ARL15*, *FST*
Low	↑Jersey	20	32.3–35.6	Exterior/conformation: *RICTOR*, *PLCXD3*, and *TTC33* Milk production: *C6*, *C7*, *DAB2*, *PLCXD3*, *RICTOR*, and *TTC33*

^1^ Significant difference in allele dosage (*p*-value < 0.05) between average allele dosage across chromosome and allele dosage at the region of selection. ^2^ Genes annotated in the CattleQTLdb (Release 49).

## Data Availability

The datasets presented in this article are not readily available because they are proprietary to Livestock Improvement Company (LIC). Requests to access the datasets should be directed to bevin.harris@lic.co.nz.

## Supplementary Materials

The following supporting information can be downloaded at: https://www.mdpi.com/article/10.3390/ani15020175/s1, Figure S1: Genomic principal component analysis of roughly equal numbers of individuals per population; Figure S2: Plot of *p*-value signal produced from XP-EHH pairwise comparison; Figure S3: Plot of *p*-value signal produced from hapFLK statistic; Figure S4: The percentage of animals to share the same ROH regions detected; Figure S5: Genomic principal component analysis using 1486 SNPs.
