# Supplementary material for: The Effect of Continuous Selection in KiwiCross® Composite Breed on Breed Ancestry and Productivity Performance
Source: Animals (Basel). 2025 Jan 10;15(2):175. doi: 10.3390/ani15020175 (PMC11758328; doi:10.3390/ani15020175)
Supplement: Supplementary file 1 [file animals-15-00175-s001.zip › animals-3399002-supplementary.pdf]

Supplementary Material

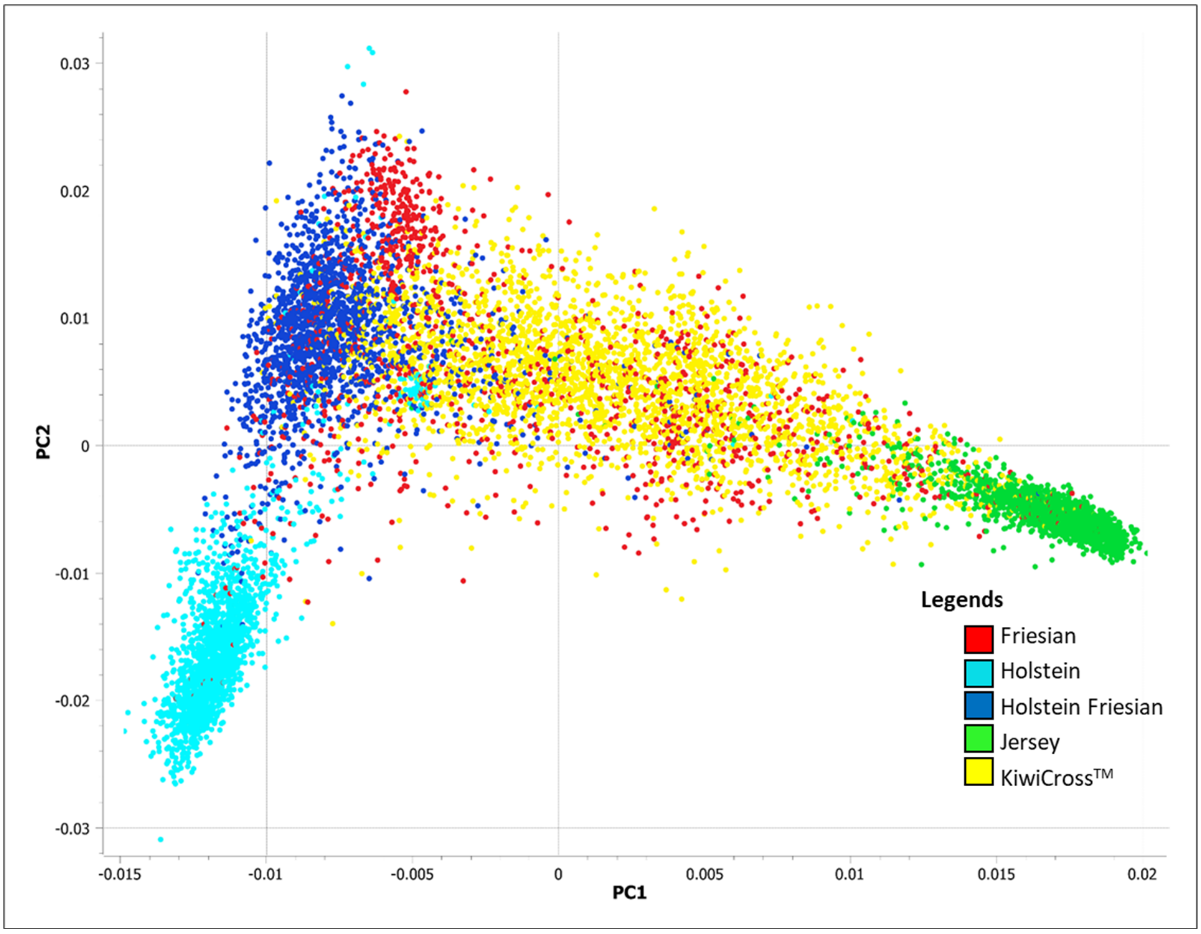

Supplemental Figure S1. Genomic principal component analysis of roughly equal numbers of individuals per population comparing the composite breed, KiwiCross® (n = 2,678) with their parental breeds, Holstein-Friesian (n = 1,748) and Jersey (n = 1,778). Purebred Holstein (n = 1,786) and Friesian (n = 1,201) were also included as the ancestry breeds for Holstein-Friesian (n = 1,748) in the analysis.

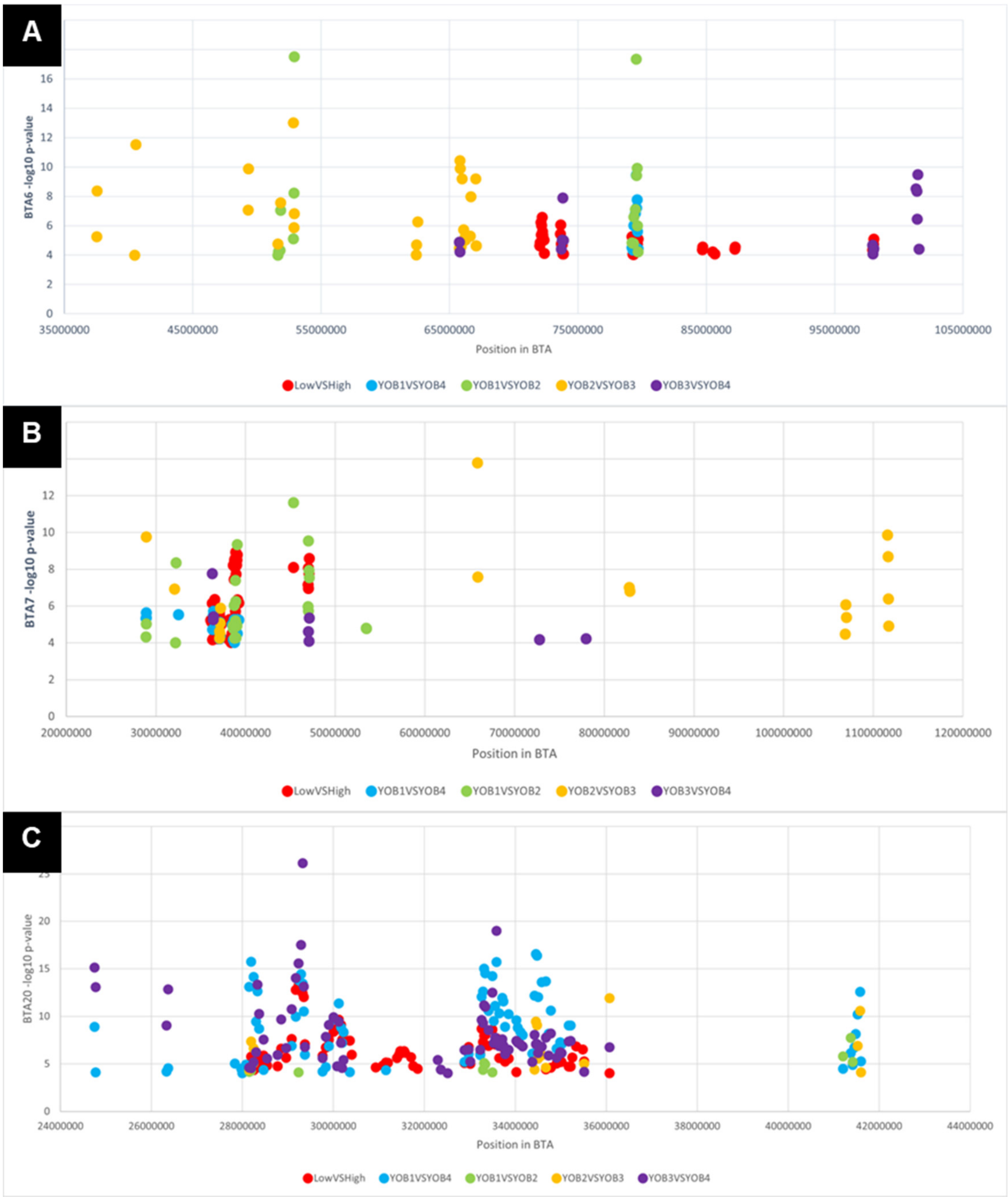

Supplemental Figure S2. Plot of p-value signal produced from XP-EHH pairwise comparison between all categories on selected region of **A)** BTA 6; **B)** BTA 7; **C)** BTA 20. ; Y-axis provides -log10 p-value of the association and X-axis provides the position of the signal on BTA. Different pairwise comparison is denoted by different colors.

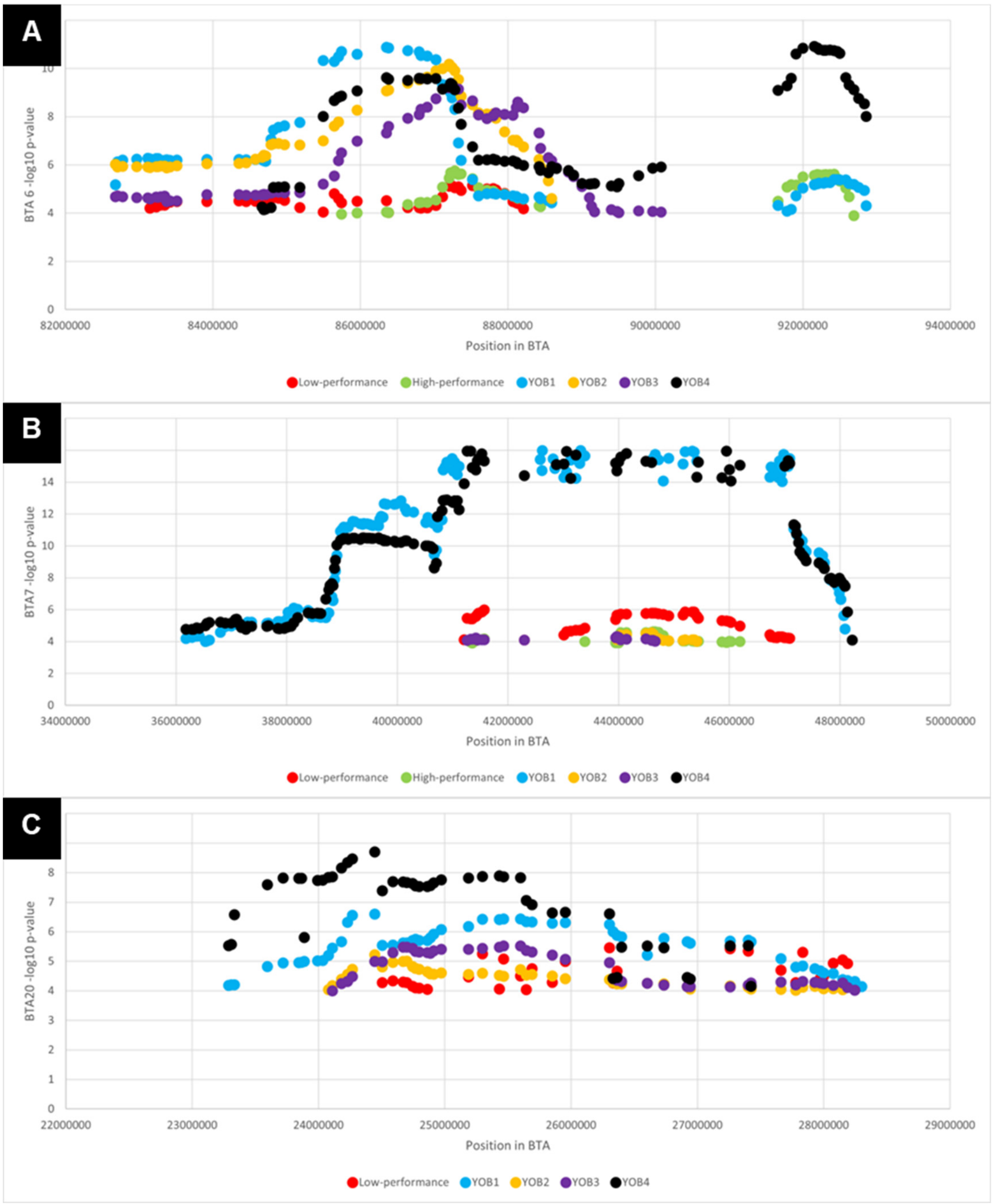

Supplemental Figure S3. Plot of p-value signal produced from hapFLK statistic within all categories on selected region of **A)** BTA 6; **B)** BTA 7; **C)** BTA 20. ; Y-axis provides -log10 p-value of the association and the X-axis provides the position of the signal on BTA. Different categories are denoted with different colors.

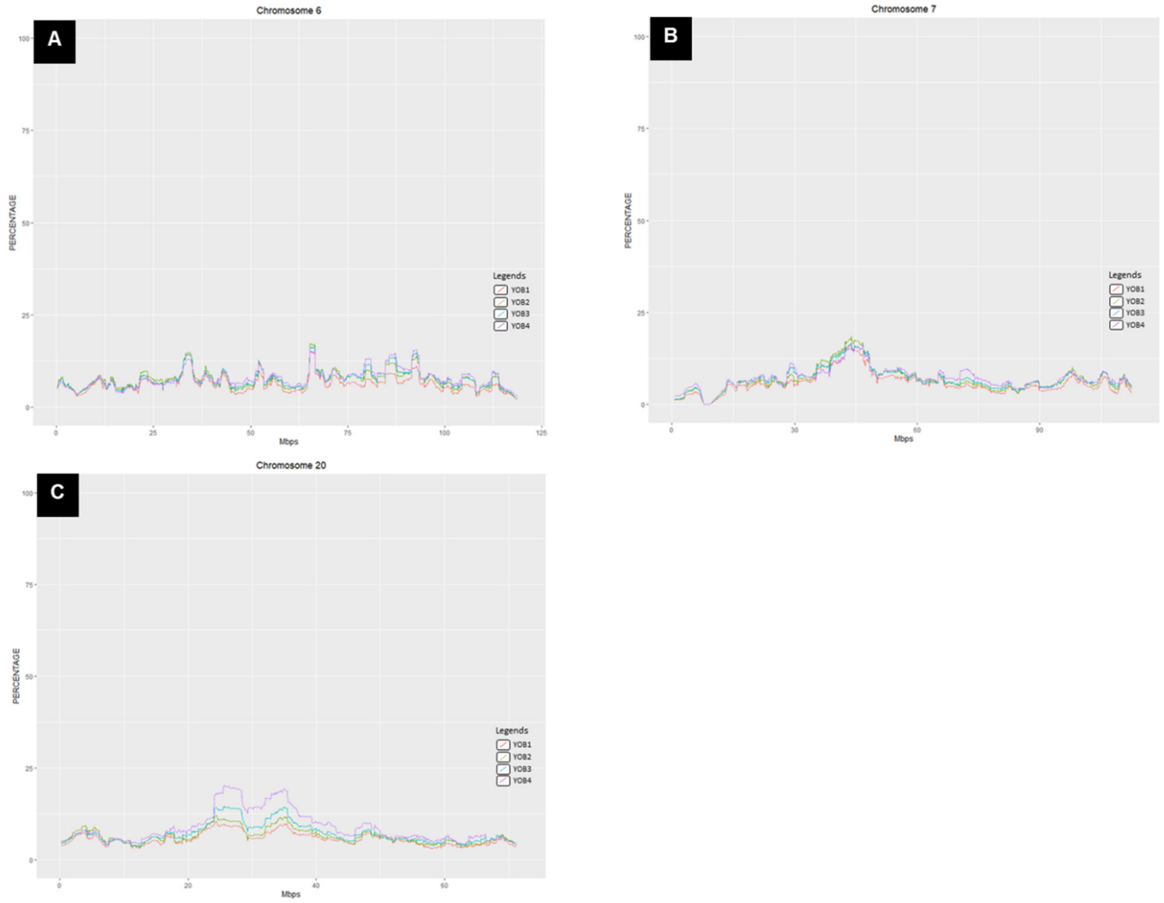

Supplemental Figure S4. The percentage of animals to share the same ROH regions detected in **A)** BTA6; **B)** BTA7; **C)** BTA20, for each YOB group depicted by a different color. Y-axis is the percentage of animals and X-axis is the position across the genome for that chromosome.
